# 88. Fibromyalgia in BehÇet's disease: any role for helicobacter screen and eradication?

**DOI:** 10.1093/rap/rky034.051

**Published:** 2018-09-20

**Authors:** Yasmin Bashir, Qainat Adamjee, C Bernard Colaço

**Affiliations:** 1 Medicine, Imperial College London, London, UNITED KINGDOM; 2 Rheumatology, Central Middlesex Hospital, London, UNITED KINGDOM

**Introduction:** Behçet’s disease (BD) is a notoriously difficult to diagnose inflammatory disorder characterised by recurrent oro-genital ulceration, often with ocular and systemic vasculitic manifestations. Diagnosis of relapsing forms may take years or decades to confirm. BD was originally described by a Turkish doctor, Hulusi Behçet in 1937, as a triad of recurrent genital and oral ulcers, as well as iritis. BD has often also been referred to as the silk-road disease, due to the particular geographical distribution of the disease along the silk route, with a greater prevalence in Turkey. Although BD is identified using the International Study Group criteria, where the presence of recurrent oral ulcers along with any two of genital ulcers, eye lesions, skin lesions and a positive pathergy test are diagnostic of BD, there is no mention of genetic markers in the criteria. However, HLA-B51 has been shown to be present in over two thirds of BD patients. Primary fibromyalgia (FM) is a non-autoimmune rheumatological disorder characterised by widespread musculoskeletal pain with no single identifiable cause but the syndrome is noted with other recognised rheumatic disorders. The diagnosis of FM is clinical. Both BD and fibromyalgia can significantly impact patients’ lives. Recently studies document a relationship between the two disorders. Clinical observations at the UK National Centre for Behçet’s Disease have suggested that there may be a higher prevalence of fibromyalgia among UK Behçet’s patients compared to the general population. Lankarani et al (2014) have reported a high prevalence of Helicobacter pylori (HP) infection in BD. We report a late diagnosis of severe BD in a patient treated as fibromyalgia, who was also infected with *H pylori*, and we document improvement following HP eradication and her subsequent BD therapy.

**Case description:** A 36-year old South Asian female patient (XX) presented at another unit in 2013 with worsening joint pains which flared during the second trimester of her third pregnancy in 2012. She first experienced mild postpartum joint pains and gastro-oesophageal reflux disease in 2010. She described the new pain as being all over, including thoracic and neck pains with shoulder and upper and lower limb pain, including the small joints of her hands, and severe left heel pain. She described swelling of small joints of her hands so she was unable to open bottles or even hold cups. This had a serious impact on her QOL as she felt exhaustion and unable to do any housework as she was in pain. It was thought that she had premature mechanical/degenerative disease and she was referred for an x-ray and a nerve conduction test, and lab tests including rheumatoid factor, vitamin D and ESR, reportedly normal. She continued to suffer with this pain for over four years as her function and quality of life deteriorated. She couldn’t do any of the housework, open bottles, open the car door, climb stairs or go shopping. She was unable to even care for her own hygiene needs, needing help to shampoo her hair and bathe herself. She became more dependent on her husband and her children. At one point she was unable to even get up from bed for about four months and remained terrified and in extreme pain. No definitive diagnosis other than fibromyalgia had been made. She was first seen in the rheumatology unit at Central Middlesex Hospital in June 2017, arriving in a wheelchair and required help for transfers to a couch. She gave a history of arthralgia as well as a new record of previous oral and genital ulcers. She was apyrexial and had palmar and extensor tenosynovitis limited lumbar flexion and extension and allodynia all over. Lab tests: HLA-B51/B58 positive and HLA-B27 negative indicated a Behçet's disorder. She was screened for *H pylori* with urea breath test and stool positive. HP eradication therapy effected a reduction of pain VAS from 10/10 to 3/10 within 4-6 weeks. Her mood and mobility improved to even allow her to play with her children and go to the park. She recommenced driving lessons. Walking time has improved dramatically. At 14-16 weeks post eradication however she noted increasing pains VAS 5 /10. She was offered colchicine 0.5 mg daily. She noted further improvement in generalised pain but at six months was relapsing with overall QOL 0% pre-diagnosis and 70% post HP eradication and colchicine was now reducing to 40% with more pain and increased depression. Repeat UBT was again strongly positive and she has undertaken secondary HP eradication with no reported complaints and further follow up is planned. With a retrospective look at her history on events taking place between 2010-2013, we recorded in 2010, becoming pregnant after a six year gap following her first child it was the happiest year of her life. In 2012, she was pregnant with her third child and it was particularly stressful as her husband was abroad and she was no family support. There was an emergency when the house flooded and she suffered a prolapse from moving furniture while pregnant. The recent flare also matched a period of her husband working away from home. Overall she is improved in her symptoms and her general wellbeing. This has been noticed by her close family and friends too who state that she definitely has a more positive attitude towards life now. She has greater independence. However, she still continues to suffer from pain, particularly in her legs, shoulders and her right wrist, which do restrict some of her activities.

**Discussion:** Diagnosis of BD depends on a range of clinical findings. Most commonly used are the International Criteria for BD (ICBD). However, despite these criteria many cases are diagnosed late. In this case, diagnosis was aided by positive HLA-B51, which has been found to increase risk of patients developing BD by factor of 5.90. XX’s severe functional symptoms were improved following just HP eradication therapy. A number of studies looked at the role of HP in BD, including Avci et al, who did not note higher prevalence of HP in BD. But in some patients, HP eradication resulted in alleviation of symptoms. In 13 BD patients in whom HP eradication was achieved, number and size of oro-genital ulcers were diminished and other manifestations of BD suppressed. XX’s further clinical response to colchicine is also supportive of diagnosis and potential impact on the inflammasome driven IL1 symptomatology. A further speculative aspect was that symptoms became more evident postpartum and after stressful events. Many patients have reported stress triggers and exacerbates BD symptoms. Studies have shown stressful life events are important in relapse and remission of BD and psychosocial factors need to be considered in management. Late diagnosis can fuel this. Not much is known about effects of pregnancy on BD, with some cases of BD flaring postpartum. A proposed mechanism for this is high levels of progesterone during pregnancy may act to attenuate the inflammatory response. In conclusion, this is a complex case of BD associated with HP which may have aggravated symptoms of systemic inflammation. Oro-genital ulceration led to immunogenetic typing and positive HLA-B51 result increased confidence of diagnosis. We suggest further large scale analyses could enable a level of importance to HP Screening in BD and the relevant contribution of HLA B51 typing may be included in the diagnostic algorithm.

**Key Learning Points:** HLA-B51 is a key genetic marker and diagnostic tool for the presence of BD and should be considered as a key diagnostic tool for BD in order to make an early diagnosis. BD has been associated with a higher prevalence of both fibromyalgia and *H pylori* (HP). HP screening and eradication therapy could prove to be beneficial in improving BD symptoms and therefore should be considered in BD patients. A key trigger for BD is stress. Late diagnosis of BD can add to the stress and potentially worsen symptoms.

**Disclosure: Y. Bashir:** None. **Q. Adamjee:** None. **C. Colaço:** None.

